# Enhanced Long‐Term Stability of Crystalline Nickel–Boride (Ni_23_B_6_) Electrocatalyst by Encapsulation with Hexagonal Boron Nitride

**DOI:** 10.1002/advs.202403674

**Published:** 2024-07-12

**Authors:** Kyung Yeol Ma, Hyeongjoon Kim, Hyuntae Hwang, Da Sol Jeong, Hoon Ju Lee, Kyeongseo Cho, Jieun Yang, Hu Young Jeong, Hyeon Suk Shin

**Affiliations:** ^1^ Department of Chemistry Ulsan National Institute of Science and Technology (UNIST) Ulsan 44919 Republic of Korea; ^2^ Department of Energy Science and Department of Chemistry Sungkyunkwan University (SKKU) Suwon 16419 Republic of Korea; ^3^ Center for 2D Quantum Heterostructures Institute of Basic Science (IBS) Sungkyunkwan University (SKKU) Suwon 16419 Republic of Korea; ^4^ Department of Chemistry and Research Institute of Basic Sciences Kyung Hee University Seoul 02447 Republic of Korea; ^5^ Graduate School of Semiconductor Materials and Devices Engineering Ulsan National Institute of Science and Technology (UNIST) Ulsan 44919 Republic of Korea

**Keywords:** catalytic transparency, electrochemical catalysts, hexagonal boron nitride, hydrogen evolution reaction, metal boride

## Abstract

Nickel boride catalysts show great potential as low‐cost and efficient alternatives to noble‐metal catalysts in acidic media; however, synthesizing and isolating a specific phase and composition of nickel boride is nontrivial, and issues persist in their long‐term stability as electrocatalysts. Here, a single‐crystal nickel boride, Ni_23_B_6_, is reported which exhibits high electrocatalytic activity for the hydrogen evolution reaction (HER) in an acidic solution, and that its poor long‐term stability can be overcome via encapsulation by single‐crystal trilayer hexagonal boron nitride (hBN) film. Interestingly, hBN‐covered Ni_23_B_6_ on a Ni substrate shows an identical overpotential of 52 mV at a current density of 10 mA cm^−2^ to that of bare Ni_23_B_6_. This phenomenon indicates that the single‐crystalline hBN layer is catalytically transparent and does not obstruct HER activation. The hBN/Ni_23_B_6_/Ni has remarkable long‐term stability with negligible changes to its polarization curves for 2000 cycles, whereas the Ni_23_B_6_/Ni shows significant degradation after 650 cycles. Furthermore, chronoamperometric measurements indicate that stability is preserved for >20 h. Long‐term stability tests also reveal that the surface morphology and chemical structure of the hBN/Ni_23_B_6_/Ni electrode remain preserved. This work provides a model for the practical design of robust and durable electrochemical catalysts through the use of hBN encapsulation.

## Graphical abstract

The single‐crystal Ni_23_B_6_ exhibits high electrocatalytic activity for the hydrogen evolution reaction (HER) in an acidic solution, but poor long‐term stability. Interestingly, hexagonal boron nitride (hBN)‐covered Ni_23_B_6_ shows high long‐term stability with an identical overpotential to that of bare Ni_23_B_6_, indicating that the single‐crystalline hBN layer is catalytically transparent and does not obstruct HER activation.

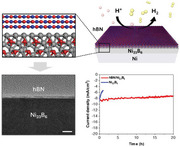

## 1 Introduction

Nickel borides are promising catalysts for a broad range of reactions, including hydrogenation, the hydrogen evolution reaction (HER), and the oxygen evolution reaction (OER).^[^ ^1^, ^2^ ^]^ However, many different nickel boride compounds exist, exhibiting various phase and chemical compositions; examples include amorphous nickel borides and crystalline NiB, Ni_3_B, Ni_2_B, Ni_4_B_3_, Ni_7_B_3_, and Ni_23_B_6_.^[^ ^3^, ^4^, ^5^ ^]^ When synthesized by heat treatment, the ultimate phase and composition of the nickel boride compounds are typically determined by reaction temperature, duration time, and cooling rate.^[^ ^3^, ^6^ ^]^ Unfortunately, most synthesis processes of nickel boride compounds generate mixed crystalline and amorphous phases. In these mixtures, the catalytic activity of the nickel borides can be varied by the ratio of the amorphous to crystalline phases; the amorphous phases are highly active catalysts for hydrogenation and other organic reactions.^[^ ^3^, ^7^ ^]^ Amorphous nickel boride and crystalline Ni_3_B have also been studied as catalysts for HER.^[^ ^5^, ^8^, ^9^ ^]^ When amorphous nickel boride is combined with metal nanoparticles, significant enhancement of the catalytic activity is observed, relative to that of the amorphous phase alone.^[^ ^8^, ^9^ ^]^ As for crystalline Ni_3_B, it is porous, and its catalytic performance is highly dependent on the amount of active surface area exposed to the electrolytes.^[^ ^5^ ^]^

Due to the easily formed product mixtures of nickel boride syntheses, the intrinsic properties of pure single‐crystalline nickel borides remain elusive.^[^ ^6^ ^]^ Among the many nickel boride phases, Ni_23_B_6_ is one of the best defined structures; it is the only face‐centered cubic (FCC) structure,^[^ ^10^ ^]^ and thus can be epitaxially formed on Ni without any structural deformation.^[^ ^11^ ^]^ Ni_23_B_6_ exhibits a metallic property that is expected to render it a good electrochemical catalyst among the metal‐rich nickel boride compounds^[^ ^12^ ^]^; however, the synthesis of Ni_23_B_6_ is limited to the undercooling method with nucleation control,^[^ ^13^ ^]^ and its applicability to catalysis is currently unknown. Furthermore, thermodynamically metastable Ni_23_B_6_ would be unstable during electrochemical reactions^[^ ^14^, ^15^ ^]^ because catalysts containing transition metals may have stability issues.^[^ ^16^ ^]^ One strategy for minimizing activity degradation during long‐term catalytic operations would be the use of encapsulation, where a protective layer is added to shield and/or passivate the catalyst from unwanted reactions while allowing (and possibly assisting) catalytic activity.

2D materials, such as graphene and hexagonal boron nitride (hBN), have been intensively considered as efficient encapsulation materials.^[^ ^17^, ^18^, ^19^, ^20^, ^21^, ^22^ ^]^ In fact, the literature includes reports of improved lifetimes for covered electrodes in a variety of electrochemical systems, including catalysts,^[^ ^23^ ^]^ molecular transport membranes,^[^ ^24^ ^]^ batteries,^[^ ^25^ ^]^ biosensors,^[^ ^26^ ^]^ and other electrochemical devices.^[^ ^27^ ^]^ Despite the negligible electrochemical catalytic activity of hBN, it has gained significant attention in investigations involving the oxygen reduction reaction (ORR), hydrogen oxidation reaction (HOR), OER, CO oxidation reaction, and methanation reaction.^[^ ^28^, ^29^, ^30^, ^31^, ^32^ ^]^ Metal catalysts for these electrochemical reactions have been reported to show improved catalytic activity and stability after they have been encapsulated by a defective layer of hBN, where reaction molecules can pass through pores, entering confined nanoreactors located between the metal and the hBN shell.^[^ ^28^, ^29^, ^30^, ^31^, ^32^ ^]^ In addition to its support functionality, hBN improved the catalytic activity of noble metals by modifying their charge‐transfer properties.^[^ ^33^, ^34^ ^]^ Ideally, highly crystalline hBN prevents the permeation of liquids and gases except for that of protons,^[^ ^35^ ^]^ but it has been shown to be transparent to atomic interactions such as van der Waals and electron‐transfer interactions.^[^ ^36^ ^]^ Such a chemically transparent barrier is attractive for the long‐term stability of electrochemical catalysts. Although theoretical and experimental studies of graphene encapsulation layers reveal them to be chemically transparent, to be able to maintain activity, and to enhance the stability of electrochemical catalysts,^[^ ^17^, ^19^ ^]^ experimental investigations of hBN encapsulation layers of high crystallinity and with minimal or no defects have not yet been reported. Unsurprisingly, there is a lack of mechanistic studies on how the defects of the encapsulating hBN affect catalysis.

In this work, we report a highly stable electrocatalyst for HER; it consists of Ni_23_B_6_ supported on a Ni substrate and encapsulated by a hBN layer (hBN/Ni_23_B_6_/Ni). Without the hBN layer, Ni_23_B_6_ is unstable in the presence of HER. The hBN‐covered Ni_23_B_6_ is formed following the growth of a single‐crystal hBN layer on Ni(111) foil using a chemical vapor deposition (CVD) method.^[^ ^11^ ^]^ Ni(111) is indeed critical to the large‐area growth of a uniform single‐crystalline hBN trilayer film. The structure of Ni(111) promotes the epitaxial growth of unidirectionally aligned hBN islands, which subsequently form a continuous film. Additionally, the significant solubility of boron in Ni substrate plays a crucial role in facilitating single‐crystalline hBN films.^[^ ^11^ ^]^ Other substrates have also been explored for the growth of large‐area single‐crystalline hBN films. For instance, liquid Au,^[^ ^22^ ^]^ Cu(110),^[^ ^37^ ^]^ and Cu(111)^[^ ^38^ ^]^ have been used. However, the hBN growth on Au and Cu substrates is typically limited to the monolayer films due to their negligible solubility for boron and nitrogen. The polarization curve and Tafel slope of hBN/Ni_23_B_6_/Ni electrodes show that catalytic activity is not impeded by the atomically thin hBN layers. Stability tests show that the hBN/Ni_23_B_6_/Ni electrodes maintain their catalytic performance after 20 h in the acidic electrolyte, indicating that the hBN/Ni_23_B_6_/Ni electrode is more stable than the uncovered Ni_23_B_6_/Ni electrode. This work demonstrates the high catalytic activity of Ni_23_B_6_ and the electrochemically transparent encapsulation effects of single‐crystalline trilayer hBN film over a metal boride electrochemical catalyst for enhanced HER stability.

## 2 Results and Discussion

### 2.1 Fabrication and Characterization of hBN/Ni_23_B_6_/Ni Electrodes

**Figure 1:**
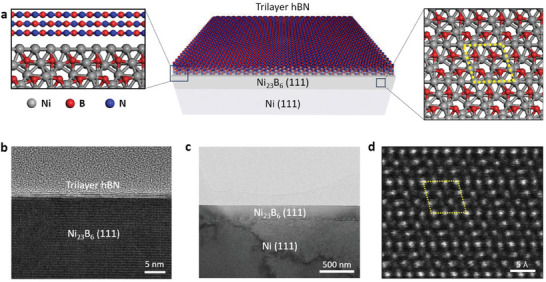
Single‐crystalline trilayer hBN film covering the Ni_23_B_6_ cathode. a) Schematic illustrations of the cathode comprising trilayer hBN/Ni_23_B_6_ layers on Ni(111) foil. Left zoomed‐in schematic shows the interface between the trilayer hBN and Ni_23_B_6_(111), including a legend for the elements present. Right zoomed‐in schematic shows the structure of the Ni_23_B_6_ layer; yellow dots outline a unit cell. b) High‐ and c) low‐magnification TEM images of the trilayer hBN/Ni_23_B_6_/Ni(111). d) Annular dark‐field STEM image of Ni_23_B_6_ at the [110] zone axis. Yellow dots indicate a unit cell of Ni_23_B_6_.

Single‐crystalline hBN encapsulating Ni_23_B_6_ on a Ni(111) substrate, abbreviated as hBN/Ni_23_B_6_/Ni, was prepared according to a previous report^[^ ^11^ ^]^ (see details in the Experimental Section). Briefly, single‐crystalline trilayer hBN films were grown on single‐crystalline Ni(111) foils over a large area using a CVD method, and during cooling, a Ni_23_B_6_ layer formed directly beneath the hBN film by the reaction of dissolved B atoms with the Ni(111) substrate. Characterization of the single‐crystal hBN films was performed after transferring them to SiO_2_ (300 nm)/Si substrates. Optical microscopy reveals uniform contrast over the entire area of the hBN film, indicating thickness uniformity over a large area (Figure S1a, Supporting Information). The line scan from atomic force microscopy (AFM) shows that the thickness of the transferred film is ≈1.2 nm (Figure S1b, Supporting Information). A typical Raman spectrum shows the *E*_2_ *_g_* mode from hBN at 1368 cm^−1^ with a full width at half‐maximum (FWHM) of ≈14 cm^–1^ (Figure S1c, Supporting Information); this is comparable to that reported for a single‐crystalline hBN monolayer.^[^ ^37^ ^]^ X‐ray photoelectron spectroscopy (XPS) measurements indicate *sp* ^2^‐hybridized B and N bonds in the hBN film. The B:N atomic ratio was found to be 1:0.96, calculated from peaks at 189.9 eV (in the binding‐energy region of B 1s) and 397.6 eV (N 1s) (Figure S1d, Supporting Information). The structure of the single‐crystalline hBN/Ni_23_B_6_/Ni is described in **Figure** **1**a, and it is consistent with the cross‐sectional transmission electron microscopy (TEM) images (Figure 1b–d). The Ni_23_B_6_ layer shows a uniform thickness of 200 nm on the surface of Ni(111) (Figure 1c) and the elemental distributions of Ni, B, and N were performed by energy‐filtered TEM imaging (Figure S2, Supporting Information). Its structure was determined using annular dark‐field scanning TEM (STEM) (Figure 1d); Ni_23_B_6_ has a face‐centered cubic structure (space group $Fm\bar{3}m$) with a lattice constant of *a* = 10.76 Å, which is three times larger than that of Ni (*a* = 3.54 Å), enabling their epitaxial relationship (Figures S3 and S4, Supporting Information).

### 2.2 HER Performance and Stability of hBN/Ni_23_B_6_/Ni Electrode

**Figure 2:**
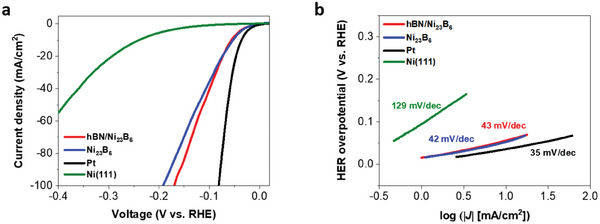
HER catalytic performance of the hBN/Ni_23_B_6_/Ni electrode and of other relevant electrodes for comparisons. a) Polarization curves and b) Tafel slopes for hBN/Ni_23_B_6_/Ni, Ni_23_B_6_/Ni, Ni(111) foil, and Pt foil electrodes measured in 0.5‐m H_2_SO_4_ at a scan rate of 5 mV s^−1^.

We evaluated the HER performance of the hBN‐covered Ni_23_B_6_ catalyst in H_2_SO_4_ solutions. To avoid exposure of the supporting Ni substrate, the edge of the electrode was masked with an electrochemically inert polytetrafluoroethylene (PTFE) adhesive tape, ensuring that the solution only made contact with hBN‐covered Ni_23_B_6_. A bias potential *V*_RHE_ (potential with respect to the reversible hydrogen electrode, RHE) was applied between the hBN/Ni_23_B_6_/Ni electrode and a graphite electrode. **Figure** **2**a shows the typical current–voltage behavior for HER. The recorded current density *j* reached −10 mA cm^−2^ at an overpotential of η of 52 mV (where η = *V*_RHE_ + 0.213 V, the theoretical equilibrium potential for water electrolysis), and it increases quickly with *V*_RHE_ at a slope of close to 42 mV dec^−1^, which is also known as the Tafel slope (Figure 2b). As a result, the *j* reaches up to −100 mA cm^−2^ at η ≈ 170 mV.

For comparison, we also measured HER performance of pristine Ni_23_B_6_/Ni, Ni(111), and Pt electrodes; to the best of our knowledge, this is the first report on the activity of Ni_23_B_6_ for HER. Pristine Ni_23_B_6_ was prepared by removing the hBN layer from hBN‐covered Ni_23_B_6_ samples using H_2_ plasma treatment (see details in the Experimental Section and Figure S5, Supporting Information). As shown in Figure 2 and **Table** **1**, the HER overpotential of Ni_23_B_6_ is also measured to be 52 mV at a current density of 10 mA cm^−1^, which is 16 mV higher than that of the Pt electrode under the same testing conditions. Interestingly, the overpotential and Tafel slopes of hBN/Ni_23_B_6_ are almost identical to those of Ni_23_B_6_, which are the best among known nickel–boride catalysts (Table S1, Supporting Information) and various 2D‐carbon and metal compound hybrid catalysts (Table S2, Supporting Information).

**Table 1:** Comparison of HER activity of hBN/Ni_23_B_6_/Ni, Ni_23_B_6_/Ni, Pt, and Ni as electrodes.

	Potential at 10 mA cm^−1^ (mV vs RHE)	Tafel slope (mV dec^−1^)	Onset potential (V)
hBN/Ni_23_B_6_/Ni	52	43	0.025
Ni_23_B_6_/Ni	52	42	0.025
Pt	36	35	0.005
Ni(111)	240	129	0.103

Our results indicate that the single‐crystalline trilayer hBN is catalytically transparent and does not obstruct HER activation. This catalytic transparency has been demonstrated in hBN‐covered Cu for the CVD of large‐area graphene^[^ ^36^ ^]^ and in a hBN‐covered OER catalyst.^[^ ^39^ ^]^ In the latter report, the catalytic activity of hBN‐covered NiFeO*_x_* H*_y_* electrodes demonstrated the electron tunneling effect through atomically thin hBN layers.^[^ ^39^ ^]^ Furthermore, the current densities *j* in electron‐tunneling devices have previously been shown to be a function of the number of hBN layers.^[^ ^40^, ^41^, ^42^ ^]^ Fast electron tunneling should be possible through trilayer hBN; it enables electron transfer between the catalyst and electrolyte to not be disturbed by the encapsulation layer, allowing its catalytic transparency. To further explore the effect of the hBN layer and its thickness dependency, we measured the HER activity of transferred trilayer hBN/Ni and as‐grown 3 nm‐thick (≈9‐layer) hBN/Ni (Figure S6 and Table S3, Supporting Information). Note that we used hBN/Ni samples instead of hBN/Ni_23_B_6_/Ni(111) in the control experiments for the effect of the hBN layer and its thickness dependency because we have not yet found the experimental conditions to grow thick hBN on Ni_23_B_6_/Ni(111). The HER activity of transferred trilayer hBN/Ni shows negligible difference compared to the bare Ni substrate, indicating that hBN itself does not exhibit significant HER activity. On the other hand, the 3 nm‐thick hBN‐covered Ni catalyst shows higher HER overpotential than bare Ni foil. This result demonstrates that relatively thick hBN degrades the catalytic activity of the Ni foil by impeding charge transfer between the Ni foil and the electrolyte. We also measured the active sites of the Ni_23_B_6_ catalyst. The effective electrochemical active surface area (ECSA) of the hBN/Ni_23_B_6_ electrode is measured to be ≈2 cm^2^ (Figure S7, Supporting Information). Furthermore, the estimated density of the surface active sites of Ni_23_B_6_ is comparable to the reported single‐crystalline Pt catalyst.^[^ ^43^ ^]^ Moreover, the electrochemical impedance spectra of Ni catalysts with and without hBN further support the no different interfacial resistance introduced by hBN encapsulation (Figure S8, Supporting Information).

**Figure 3:**
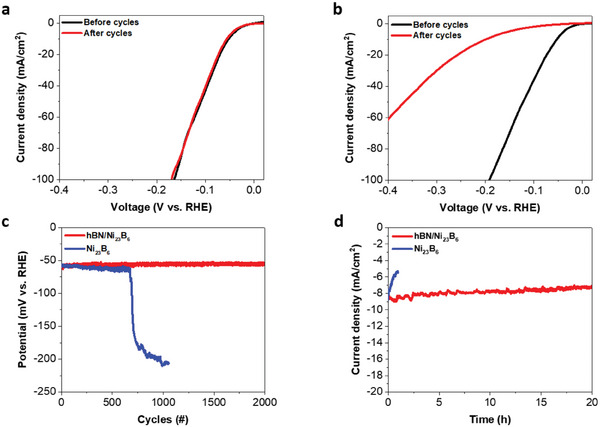
Electrochemical stability tests of the hBN/Ni_23_B_6_/Ni and Ni_23_B_6_/Ni electrodes. (a,b) Polarization curves before (black curve) and after (red) 2000 cycles for the a) hBN/Ni_23_B_6_/Ni and b) Ni_23_B_6_/Ni electrodes. c) Results of a long‐term stability test of Ni_23_B_6_ with and without hBN. d) Chronoamperometry measurements of the hBN/Ni_23_B_6_/Ni and Ni_23_B_6_/Ni electrodes under an overpotential of 52 mV.

Along with electrocatalytic transparency, we found that the hBN encapsulation layer blocks the access of reactive species to the Ni_23_B_6_ surface, preventing degradation. Investigations of the electrochemical stability of the hBN/Ni_23_B_6_/Ni and Ni_23_B_6_/Ni electrodes were carried out over 2000 cycles. The high current density of the hBN/Ni_23_B_6_/Ni electrode shows remarkable long‐term stability with negligible differences in the polarization curves and the same overpotential even after 2000 cycles (**Figure** **3**a,c). In contrast, the Ni_23_B_6_/Ni electrode shows noticeable degradation after 650 cycles (Figure 3c). After 2000 cycles, the overpotential degrades significantly to ≈200 mV at a current density of 10 mA cm^−1^ (Figure 3b), similar to the reference Ni sample (Figure 2a, green). XPS indicates that the peak for the Ni─B bond in the B 1s spectrum disappears after 650 cycles (Figure S9, Supporting Information); we attribute this to the 200 nm‐thick Ni_23_B_6_ catalyst layer being etched by the highly reactive environment of the acidic electrolyte. To further investigate the protective effect of hBN, we performed chronoamperometry (CA) studies on the hBN/Ni_23_B_6_/Ni and Ni_23_B_6_/Ni electrodes (Figure 3d). CA evaluates the long‐term stability of the current density at a constant potential. Under an overpotential of ≈52 mV, the current density of Ni_23_B_6_ quickly decayed to only ≈67.5% of its original value after 1 h. In contrast, the current density of the hBN/Ni_23_B_6_ catalyst under the same conditions was maintained at ≈95% for 20 h. The enhanced catalytic stability of hBN/Ni_23_B_6_ during the CA test can be attributed to a passivation effect that prevents the catalyst from degradation.

**Figure 4:**
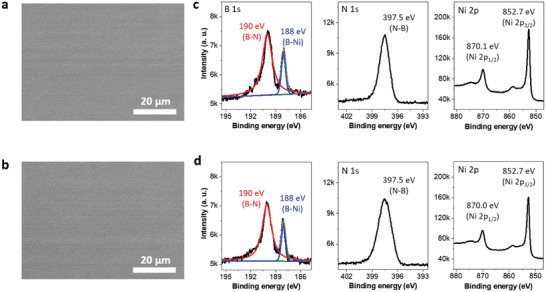
Stability of hBN/Ni_23_B_6_/Ni electrodes with respect to HER. (a,b) SEM images of hBN/Ni_23_B_6_ surface a) before and b) after HER. c) XPS of the as‐fabricated hBN/Ni_23_B_6_/Ni electrode. The two peaks in the binding‐energy region of B 1s appear at 190.0 eV (B─N) and 188.0 eV (B─Ni). In the N 1s region, one peak appears at 397.5 eV (N─B). The Ni 2p spectrum shows metallic Ni peaks at 870.1 eV and 852.7 eV. d) XPS spectra of the hBN/Ni_23_B_6_/Ni electrode after HER tests, revealing the electrode did not change.

To evaluate whether the morphology of hBN/Ni_23_B_6_ changes during the reaction in acidic electrolytes, we evaluated the surface morphology before and after HER using scanning electron microscopy (SEM; **Figure** **4**a,b). Uniform contrast is revealed on the hBN surface before and after HER for the hBN/Ni_23_B_6_ sample, indicating that the entire Ni_23_B_6_ surface is covered by hBN even after HER. We then used XPS to define the chemical bonding structure of the hBN/Ni_23_B_6_ before the reaction. The spectrum in the binding‐energy region of B 1s was deconvoluted into two distinct peaks at 190 and 188 eV, which correspond to the B─N and B─Ni bonds, respectively (Figure 4c). The N 1s spectrum shows a single peak, indicating that only N with N─B bonds are present. All XPS spectra show negligible differences between those before and after HER stability tests (Figure 4d). These experimental results suggest that the hBN encapsulation of the electrochemical catalyst allows the realization of long‐term stability within acidic electrolytes (0.5 m H_2_SO_4_), making them potentially useful to various electrode applications.

According to a recent report, even hBN encapsulation involving a *mono*layer film can enhance the efficiency of the OER and long‐term stability.^[^ ^39^ ^]^ This report demonstrated the protective effects of hBN encapsulation through many control experiments; however, the encapsulation layer was transferred from a growth substrate, and the transfer process (physical delamination) may cause many defects such as cracks, holes, and folds. The direct growth of hBN onto electrochemical catalysts, such as in the preparation of hBN/Ni_23_B_6_/Ni, avoids such damage, but direct growth typically requires high temperatures that are well above ≈1000 °C; therefore, it is desirable for future efforts to develop a versatile method capable of growing highly crystalline hBN layers on various electrochemical catalysts for practical applications.

In summary, we have presented both HER electrocatalytic activity and stability of Ni_23_B_6_ in an acidic solution. Its poor HER stability was highly improved through its encapsulation by a hBN layer, which acts as an electrocatalytic transparent layer, preserving the HER activity of Ni_23_B_6_. Electrochemical long‐term stability tests show a maintained polarization curve for 2000 cycles and a stable chronoamperometric curve for 20 h. Furthermore, the morphology and chemical structure are maintained after long‐term HER tests. Our results indicate that the hBN encapsulation can be utilized to dramatically enhance the stability of electrochemical catalysts without performance degradation.

## 3 Experimental Section

### Preparation of Single‐Crystalline Ni(111) Foil

The Ni(111) foil was prepared by contact‐free annealing. A suspended commercial polycrystalline Ni foil (100 µm thick, 99.994%) was heated to 1350 °C, and held at that temperature for 24 h under a mixed‐gas flow (Ar, 30 sccm; H_2_, 30 sccm; sccm, standard cubic centimeters per minute) at a pressure of 760 torr.

### Fabrication of Ni_23_B_6_ Cathode Covered by Single‐Crystalline Trilayer hBN, hBN/Ni_23_B_6_/Ni

The annealed Ni(111) foil was placed inside a high‐temperature and low‐pressure CVD system at the center of a furnace. A borazine precursor flask was placed in a bath of ethylene glycol and water at −15 °C. The bath temperature was ramped up to 25 °C, and the Ni(111) foil was heated to a growth temperature of 1220 °C under a mixed‐gas flow (Ar, 10 sccm; H_2_, 10 sccm). The flow of borazine gas at 0.1 sccm (controlled by a mass flow controller) was then introduced into the CVD chamber for 60 min. After hBN growth, the borazine flow was terminated, and the furnace was cooled rapidly to room temperature under a mixed‐gas flow (Ar, 10 sccm; H_2_, 10 sccm). Ni_23_B_6_ forms during the cool down, resulting in hBN/Ni_23_B_6_/Ni.

### Transfer of hBN Films onto Arbitrary Substrates

The as‐grown hBN was transferred from Ni_23_B_6_/Ni to SiO_2_/Si wafers by a polymer‐mediated wet‐transfer process for further characterization. In this process, a poly(methyl methacrylate) (PMMA) film was spin‐coated on the as‐grown hBN/Ni_23_B_6_/Ni as a protective layer. Then, Ni_23_B_6_/Ni was etched away in an aqueous solution of iron chloride (FeCl_3_). After thorough washing, the hBN film was transferred to the target substrate, and finally, the PMMA was removed by dipping it in acetone.

### Removal of the hBN Layer from the hBN/Ni_23_B_6_/Ni Sample using H_2_ Plasma Etching

To evaluate the electrocatalytic performance of Ni_23_B_6_ without the protective hBN layer, the as‐grown hBN/Ni_23_B_6_/Ni sample was exposed to 50 W of H_2_ plasma using a 13.56‐MHz radio‐frequency plasma generator (Diener Femto, Diener Electronic) for 5 min. The sample was kept 10 cm away from the discharge zone. Plasma treatment was performed at 25 °C with an H_2_ gas flow (5 sccm). The operating pressure was maintained at 1 mbar during plasma exposure. No potential was applied to the aluminum stage on which the sample was placed.

### Characterization Techniques

The prepared Ni(111) foil was characterized by SEM (Verios 460, FEI) and electron‐backscatter diffraction (Hikari from Ametek). The surface morphology of hBN was characterized by optical microscopy (Axio Scope. A1, Carl Zeiss), SEM (Verios 460, FEI), and AFM (Dimension Icon, Bruker). Typical conditions of 5.0 kV and 0.8 µA were adapted for all SEM images. XPS (ESCALAB 250 Xi, Thermo Scientific) was performed to determine chemical compositions. Note that the 20.0 eV of pass energy and 0.05 eV of energy step size were adapted for the precise XPS measurements. Raman spectra were obtained using a micro‐Raman spectrometer (alpha 300, WITec GmBH) with a laser excitation wavelength of 532 nm and power of ≈2 mW. For atomic‐resolution TEM and STEM imaging, a spherical aberration (Cs)‐corrected TEM (Titan^3^ G2 60–300, FEI) was used at 80 and 200 kV, respectively.

### Electrochemical Measurements

Electrochemical measurements were performed in a three‐electrode cell using a ZIVE SP2 (ZIVE LAB). Polarization curves were collected by linear‐sweep voltammetry with a scan rate of 5 mV s^−1^ in 0.5 m H_2_SO_4_ electrolyte. A square metal‐foil electrode of geometric area 1 cm × 1 cm was used as the working electrode and set up in a customized holder for measurements. Ag/AgCl and graphite rod were used as the reference electrode and counter electrode, respectively. During electrochemical measurements, high‐purity N_2_ gas was continuously bubbled. Potentials versus RHE can be calculated and compared with the Ag/AgCl electrode by adding a value of 0.215 V after calibration. The electrochemical stabilities of the catalysts were evaluated by continuously cycling the catalyst at a scan rate of 5 mV s^−1^, for up to 2000 cycles. The chronoamperometry measurements were done after stabilization for 7 h.

## Conflict of Interest

The authors declare no conflict of interest.

## Author Contributions

H.S.S. and K.Y.M. conceived the project. K.Y.M. grew the hBN and Ni_23_B_6_ by CVD, fabricated the electrochemical electrodes, and carried out optical microscopy, SEM, AFM, and Raman measurements. H.K. and H.H. carried out XPS measurements. K.Y.M., H.H., D.S.J., H.J.L., K.C., and J.Y. performed electrochemical measurements. H.Y.J. performed TEM and STEM measurements. H.S.S supervised this project. All authors contributed to writing of the manuscript.

## Supporting information

Supporting Information

## Data Availability

The data that support the findings of this study are available from the corresponding author upon reasonable request.
