# Supplementary material for: Enhanced Long‐Term Stability of Crystalline Nickel–Boride (Ni23B6) Electrocatalyst by Encapsulation with Hexagonal Boron Nitride
Source: Adv Sci (Weinh). 2024 Jul 12;11(35):2403674. doi: 10.1002/advs.202403674 (PMC11425285; doi:10.1002/advs.202403674)
Supplement: Supplementary file 1 — Supporting Information [file ADVS-11-2403674-s001.docx]

**Supporting Information for**

**Enhanced Long-Term Stability of Crystalline Nickel Boride (Ni_23_B_6_) Electrocatalyst by Encapsulation with Hexagonal Boron Nitride**

Kyung Yeol Ma^1,2,3^, Hyeongjoon Kim^1^, Hyuntae Hwang^1^, Da Sol Jeong^1^, Hoon Ju Lee^2,3^, Kyeongseo Cho^2,3^, Jieun Yang^4^, Hu Young Jeong^5^, and Hyeon Suk Shin^1,2,3,5,^*

^1^Department of Chemistry, Ulsan National Institute of Science and Technology (UNIST), Ulsan 44919, Republic of Korea

^2^Department of Energy Science and Department of Chemistry, Sungkyunkwan University (SKKU), Suwon 16419, Republic of Korea

^3^Center for 2D Quantum Heterostructures, Institute of Basic Science (IBS), Sungkyunkwan University (SKKU), Suwon 16419, Republic of Korea

^4^Department of Chemistry and Research Institute of Basic Sciences, Kyung Hee University, Seoul 02447, Republic of Korea

^5^Graduate School of Semiconductor Materials and Devices Engineering, Ulsan National Institute of Science and Technology (UNIST), Ulsan 44919, Republic of Korea

*Corresponding author: shin0902@skku.edu (H.S.S.)


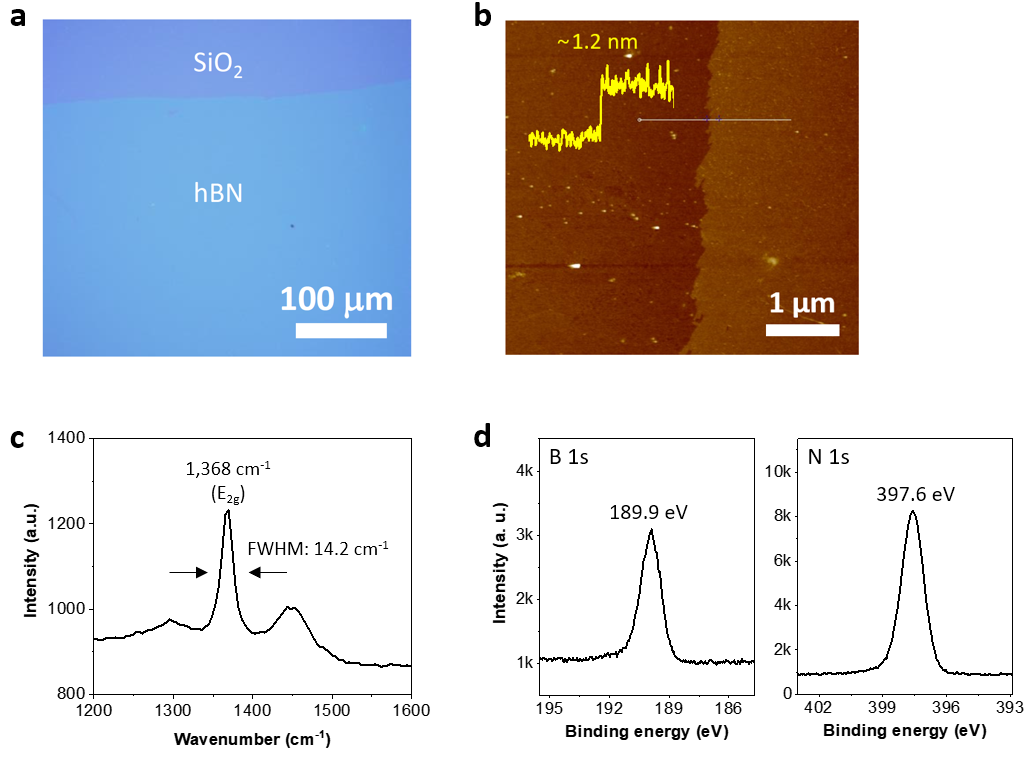


**Figure S1. Characterization of hBN film after its wet-transfer to a SiO_2_ (300 nm)/Si substrate.** (a) Optical microscopy image of the film, showing its uniformity. (b) AFM image of the trilayer hBN; inset line scan shows the height profile. (c) Raman spectrum of the trilayer hBN film. FWHM is estimated to be 14.2 cm^–1^, indicating high-quality crystallinity. (d) XPS spectra of the hBN film in the binding-energy regions of B 1s and N 1s.


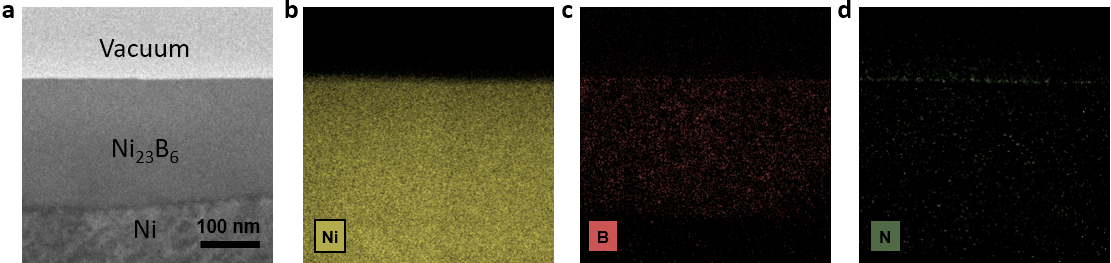

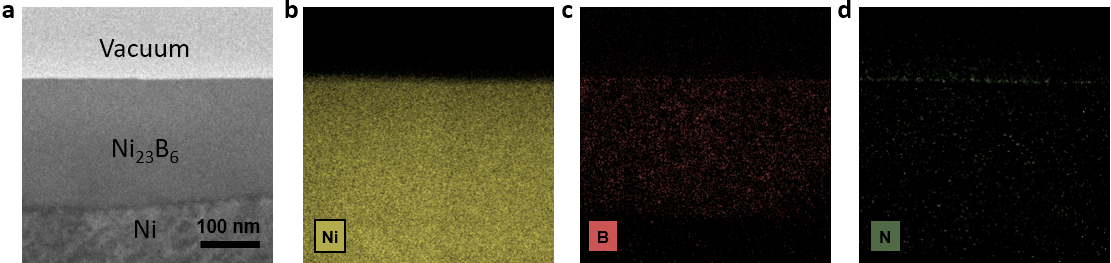


**Figure S2. Energy-filtered (EF) TEM mapping of hBN/Ni_23_B_6_/Ni electrode.** (a) Cross-sectional TEM image. (b) EF-TEM maps of Ni (yellow), (c) boron (red), and (d) nitrogen (green).


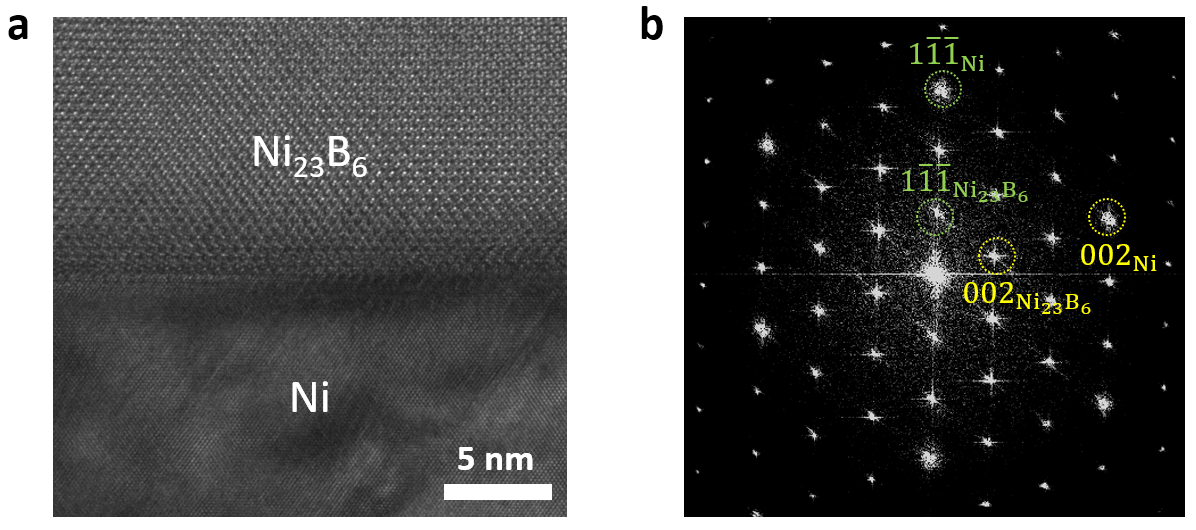


**Figure S3. Epitaxial relationship between Ni_23_B_6_ and Ni(111).** (a) High-magnification TEM image of the Ni_23_B_6_/Ni interface. (b) Fast Fourier transform (FFT) pattern for the Ni_23_B_6_ and Ni interface.


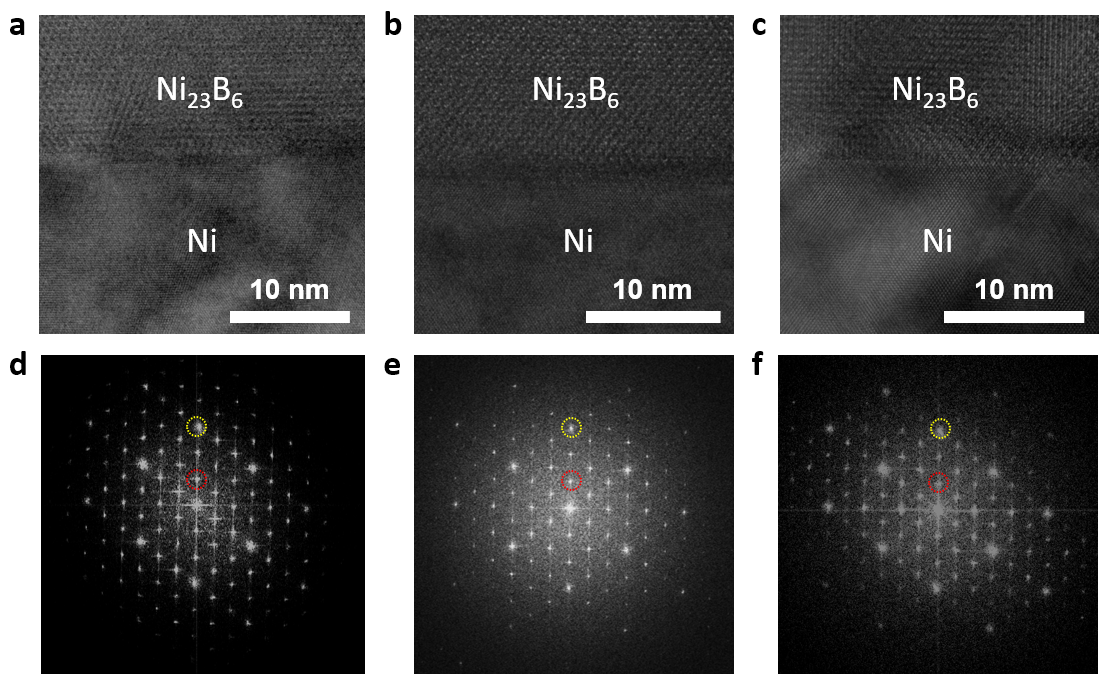


**Figure S4. Epitaxial relationship between Ni_23_B_6_ and Ni(111) at multiple positions.** (a-c) TEM images of the Ni_23_B_6_/Ni(111) interface at multiple positions. (d-f) Fast Fourier transform (FFT) images of (a-c) Ni_23_B_6_ and Ni(111) interfaces. Yellow and red dashed circles indicate Ni_23_B_6_ and Ni, respectively.


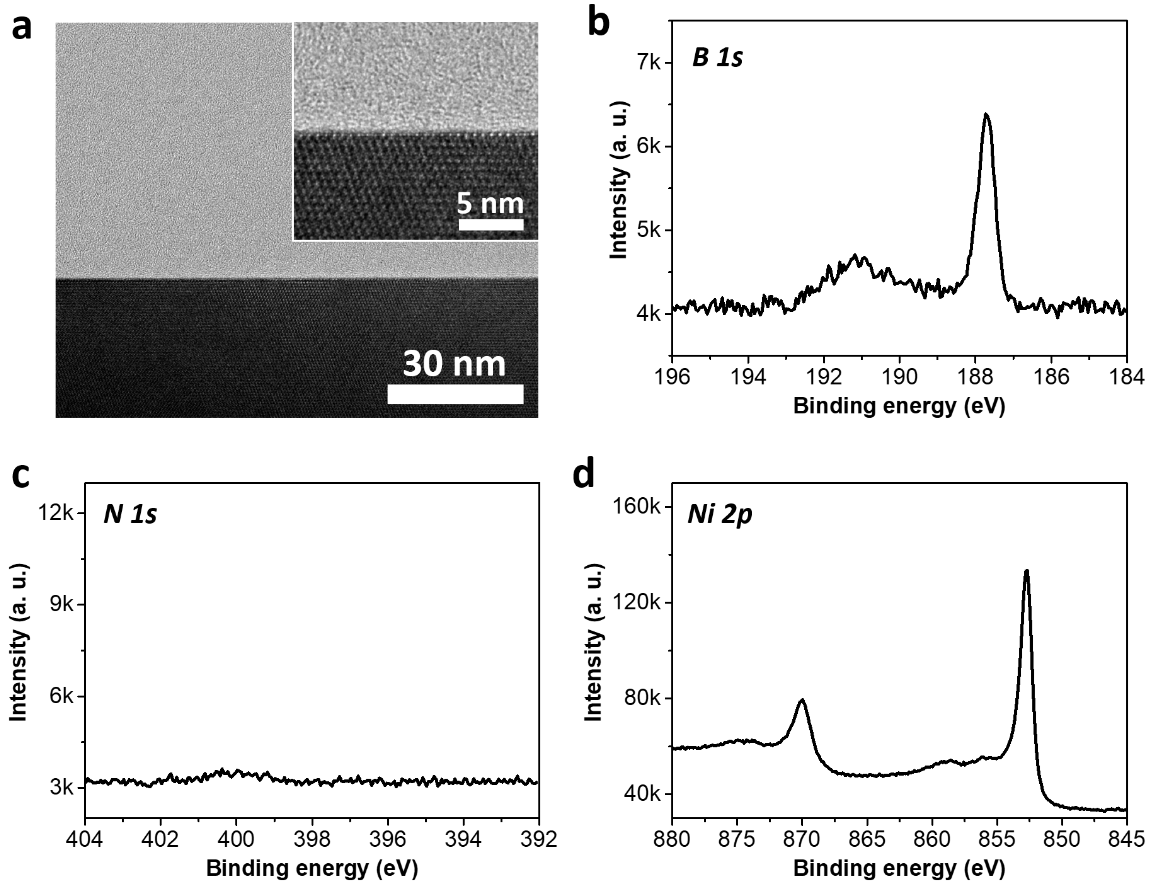


**Figure S5. Characterization of pristine Ni_23_B_6_ layer.** hBN is removed by exposing hBN/Ni_23_B_6_/Ni to 50 W of H_2_ plasma treatment for 5 min. (a) Cross-sectional TEM images of Ni_23_B_6_ without the protective hBN layer. (b–d) XPS spectra showing the binding-energy regions of (b) B 1s, (c) N 1s, and (d) Ni 2p. The weak and broad peak at 191.1 eV in the B 1s spectrum is attributed to residual BN.


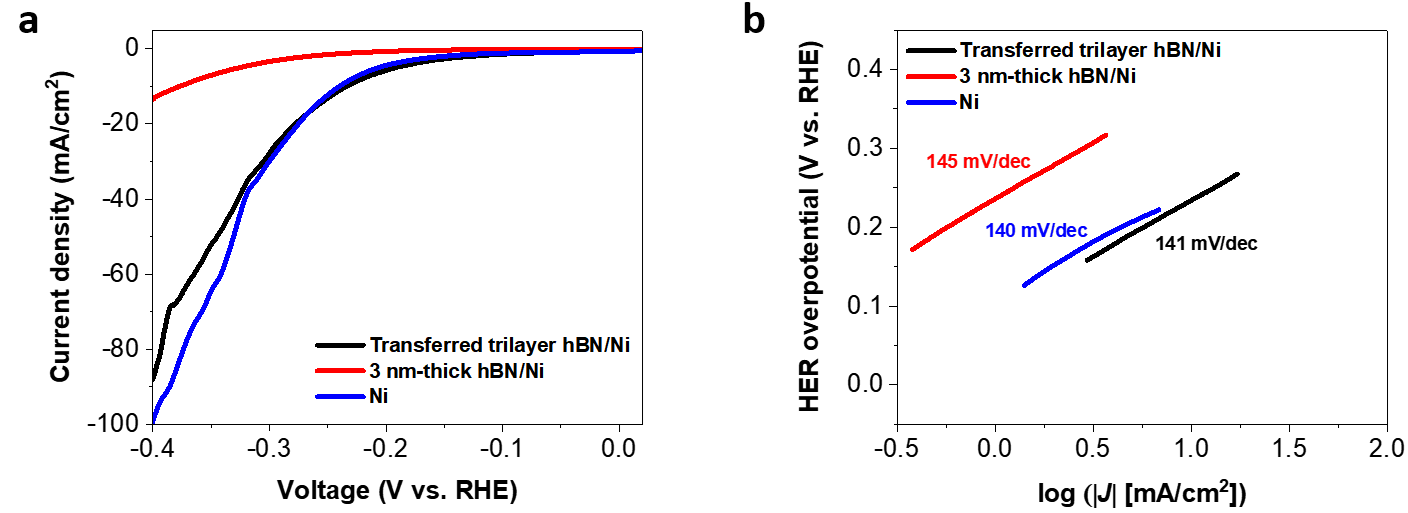


**Figure S6. HER catalytic performance of the transferred trilayer hBN/Ni, 3 nm-thick (~9-layer) hBN/Ni, and Ni electrodes.** (a) Polarization curves and (b) Tafel slopes for transferred trilayer hBN/Ni, 3 nm-thick hBN/Ni, and Ni electrodes measured in 0.5 M H_2_SO_4_ with a scan rate of 5 mV s^-1^.


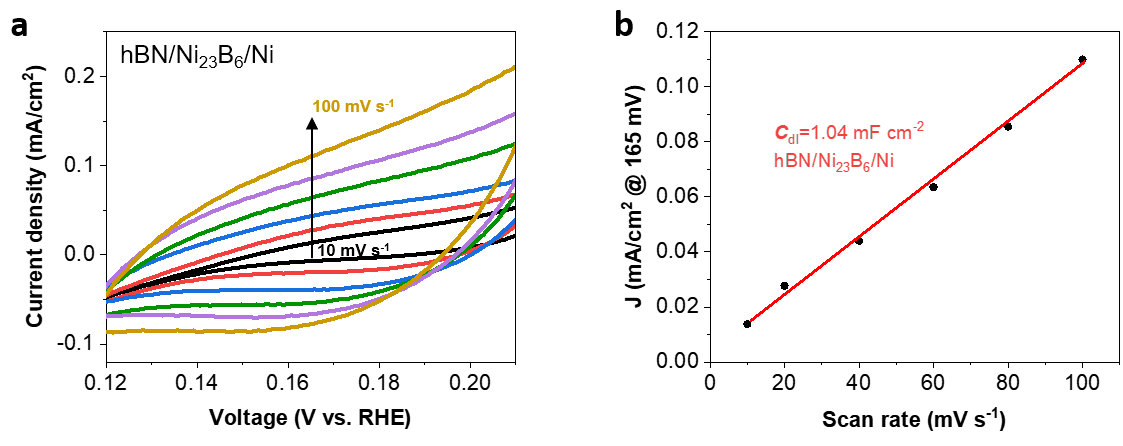


**Figure S7. Electrochemical active surface area (ECSA) and density of the surface active sites calculations.** (a) Cyclic voltammetry (CV) curves for hBN/Ni_23_B_6_/Ni carried out in non-faradic regions at different scan rates in 0.5 M H_2_SO_4_. (b) The *C*_dl_ calculations. By plotting the difference of current density (*J*) between the anodic and cathodic sweeps (*J*_anodic_-*J*_cathodic_)/2(at 0.165 V versus RHE) against the scan rate, a linear trend was constructed with its slope the double-layer capacitances C_dl_ (1.04 mF cm^-2^). The ECSA of catalyst is estimated from the C_dl_ according to Equation: *ECSA = (C_dl_/C_s_)×A_geometry_*, where Cs (0.124 mF cm^-2^) is specific capacitance of our Ni foil, A_geometry_ (0.25 cm^2^) is the geometric surface area of the catalyst electrodes. The effective ECSA of the hBN/Ni_23_B_6_/Ni is measured to be 2.09 cm^2^. Furthermore, the density of active sites is determined by the lattice constant (Figure 1d, ADF-STEM image). The surface area of the unit cell is $6.95\times{10}^{-14}$ cm^2^. We assumed that the entire (111) basal plane can be catalytically active. Therefore, the density of active sites is estimated to be about $3.59\times{10}^{15}$ cm^-2^. The density of the surface active sites of Ni_23_B_6_ on the geometric area is $3.59\times{10}^{15}$ sites cm^-2^ $\times$ 2.09 $=$ $7.50\times{10}^{15}$ sites cm^-2^.


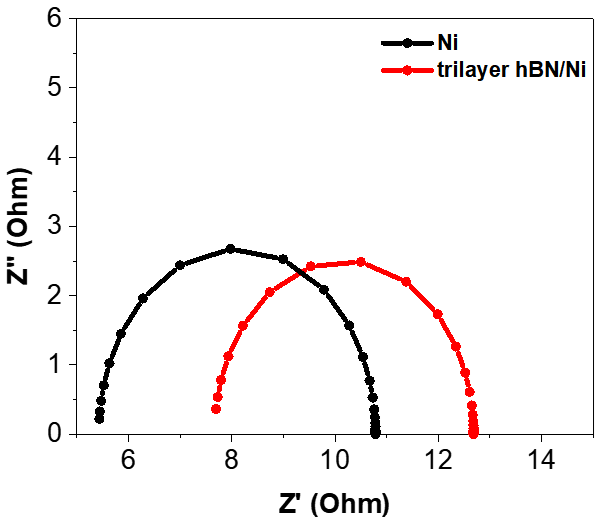


**Figure S8. Electrochemical impedance spectroscopy.** (a) Nyquist plots of trilayer hBN/Ni and Ni. The plots were fitted using an equivalent circuit to extract the series and charge transfer resistances. The axes labeled Z’ and Z’’ are real and imaginary, respectively. The charge transfer resistance of 3-layer hBN/Ni (4.99 Ω/cm^2^) is comparable to that of Ni (5.35 Ω/cm^2^) at -0.6 V versus RHE, which indicates a negligible difference of interlayer charge transfer impedance. Impedance measurements were performed from 0.1 Hz to 0.2 MHz with a 10 mV AC amplitude.


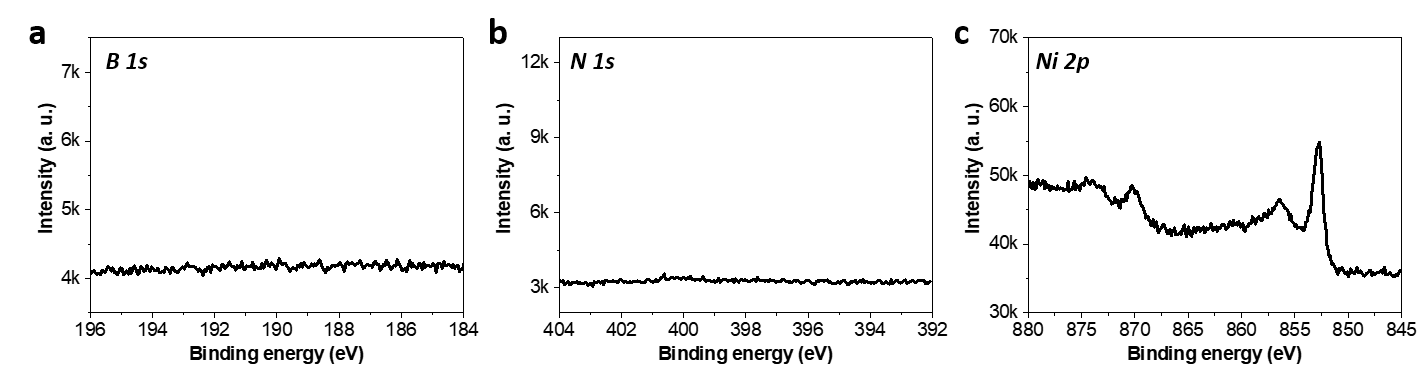


**Figure S9. XPS of Ni_23_B_6_ sample after long-term electrochemical stability test.** X-ray photoelectron spectra of a Ni_23_B_6_ sample after 650 cycles showing the binding-energy regions of (a) B 1s, (b) N 1s, and (c) Ni 2p.

**Table S1. Comparison of HER activity of nickel–boride catalysts in acidic electrolytes**

NPs: nanoparticles; GCE: glassy carbon electrode

|  | **Electrolyte** | **Potential at 10 mA/cm^2^ (mV vs RHE)** | **Tafel slope  (mV dec^-1^)** | **References** |
| --- | --- | --- | --- | --- |
| hBN/Ni_23_B_6_ | 0.5 M H_2_SO_4_ | 52 | 42 | This work |
| Ni_23_B_6_ | 0.5 M H_2_SO_4_ | 52 | 43 | This work |
| Amorphous Ni-B_0.54_ @Ni NPs | 0.5 M H_2_SO_4_ | 45 | 43 | [1] *Nano Energy* **2016**, 19, 98 |
| Porous Ni_3_B | 0.5 M H_2_SO_4_ | 79 | 85.32 | [2] *Appl. Surf. Sci.* **2019**, 470, 591 |
| Ni_4_B_3_ | 0.5 M H_2_SO_4_ | 182 | 114.7 | [2] *Appl. Surf. Sci.* **2019**, 470, 591 |
| Ni_2_B | 0.5 M H_2_SO_4_ | 252 | 121.3 | [2] *Appl. Surf. Sci.* **2019**, 470, 591 |
| Ni-Co-Mo-B | 0.5 M H_2_SO_4_ | 145 | - | [4] *Nano Energy* **2020**, 67, 104245 |
| Ni_3_B/MoB | 0.5 M H_2_SO_4_ | 75 | 61 | [5] *Chem. Eng. J.* **2021**, 405, 126977 |
| Ni_3_B | 0.5 M H_2_SO_4_ | 167 | 155 | [5] *Chem. Eng. J.* **2021**, 405, 126977 |
| Amorphous Ni-B_2.7_ NPs on GCE | 0.1 M HClO_4_ | ≒170 | - | [6] *ChemCatChem* **2016**, 8, 708 |

**Table S2. Comparison of HER activity of various 2D-carbon and metal compound hybrid catalysts in acidic electrolytes**

|  | Electrolyte | Potential at 10 mA/cm^2^ (mV vs RHE) | Tafel slope  (mV dec^-1^) | References |
| --- | --- | --- | --- | --- |
| hBN/Ni_23_B_6_/Ni | 0.5 M H_2_SO_4_ | 52 | 42 | This work |
| WO_x_@C/C | 0.5 M H_2_SO_4_ | 15 | 19.17 | [1] *Adv. Mater.* **2018**, 30, 1705979 |
| MoS_2_/Graphene | 0.5 M H_2_SO_4_ | 110 | 67.4 | [2] *Chem. Mater.* **2016**, 28, 5733. |
| MoS_2_/MoP/N-doped carbon inner layer | 0.5 M H_2_SO_4_ | 151 | 58 | [3] *J. Mater. Chem. A* **2018**, 6, 24783. |
| Carbon paper@TiO_2_@MoS_2_ | 0.5 M H_2_SO_4_ | 188  (at 20 mA/cm^2^) | 41.7 | [4] *ACS Appl. Mater. Interfaces* **2018**, 10, 6084. |
| Co@BCN | 0.5 M H_2_SO_4_ | 96 | 63.7 | [5] *ACS Nano* **2016**, 10, 684. |
| N-Co@Graphene | 0.5 M H_2_SO_4_ | 265 | 98 | [6] *ACS Appl. Mater. Interfaces* **2016**, 7, 8083. |
| Graphene-coated Cu NWs | 0.5 M H_2_SO_4_ | 252 | 67 | [7] *J. Mater. Chem. A* **2017**, 5, 13320. |

**Table S3. Comparison of HER activity of transferred trilayer hBN/Ni, 3 nm-thick (~9 layer) hBN/Ni , and bare Ni foil as electrodes**

|  | **Potential at 10 mA cm^-1^ (mV vs. RHE)** | **Tefel slope (mV dec^-1^)** |
| --- | --- | --- |
| Transferred trilayer hBN/Ni | 238 | 141 |
| 3 nm-thick (~9-layer) hBN/Ni | 378 | 145 |
| Ni | 240 | 140 |
